# Management and outcomes of severe dengue patients presenting with sepsis in a tropical country

**DOI:** 10.1371/journal.pone.0176233

**Published:** 2017-04-24

**Authors:** Prapit Teparrukkul, Viriya Hantrakun, Nicholas P. J. Day, T. Eoin West, Direk Limmathurotsakul

**Affiliations:** 1 Medical department, Sunpasithiprasong Hospital, Ubon Ratchathani, Thailand; 2 Mahidol-Oxford Tropical Medicine Research Unit, Faculty of Tropical Medicine, Mahidol University, Bangkok, Thailand; 3 Centre for Tropical Medicine and Global Health, Nuffield Department of Medicine, University of Oxford, Oxford, United Kingdom; 4 Division of Pulmonary and Critical Care Medicine, Department of Medicine, University of Washington, Seattle, Washington, United States of America; 5 Department of Global Health, University of Washington, Seattle, Washington, United States of America; 6 Department of Microbiology and Immunology, Faculty of Tropical Medicine, Mahidol University, Bangkok, Thailand; 7 Department of Tropical Hygiene, Faculty of Tropical Medicine, Mahidol University, Bangkok, Thailand; Institute of Tropical Medicine (NEKKEN), Nagasaki University, JAPAN

## Abstract

**Background:**

Dengue is a common cause of infection in adults in tropical countries. Sepsis is a syndrome of systemic manifestations induced by infection of any organisms; including bacterial, fungal and viral agents. Here, we investigated the diagnosis, management and outcomes of dengue patients presenting with sepsis in a prospective study of community-acquired sepsis in Thailand.

**Methods:**

From June to December 2015, 874 adult patients (age≥18 years) with suspected or documented community-acquired infection, with ≥3 diagnostic criteria for sepsis according to the Surviving Sepsis Campaign 2012, and within 24 hours of admission were evaluated. Serum was stored and later tested for dengue PCR assays.

**Results:**

A total of 126 patients had dengue PCR assays positive (2 DENV-1, 12 DENV-2, 24 DENV-3 and 88 DENV-4), and 5 of them (4%) died. We found that attending physicians suspected dengue infection on admission in 84 patients (67%), and recorded dengue infection as the final diagnosis in 96 patients (76%). Four of five fatal cases were diagnosed and treated as septic shock not due to dengue. In multivariable analysis, there was a trend showing that age≥60 years, hypoxemia and misdiagnosis of dengue by attending physicians were associated with 28-day mortality.

**Conclusions:**

A number of adult patients who died of dengue are misdiagnosed as severe sepsis and septic shock. Diagnosis of dengue based on clinical features alone is difficult. Rapid diagnostic tests for dengue may need to be routinely used in adult patients presenting with sepsis and septic shock in tropical countries. This approach could improve diagnosis and management of those patients.

## Introduction

Dengue is a common cause of fever in adult patients in tropical countries. A prospective study in Laos shows that 9% (112/1215) of adult patients (age>15 years) presenting with non-malarial fever have dengue [1]. A prospective study in Papua Indonesia similarly demonstrates that about 5% of adult patients (age>18 years) presenting with non-malarial fever have dengue [2]. These figures are probably representative of all tropical countries in Asia, Africa and the Americas where dengue is endemic [3, 4].

Classic dengue or “break bone fever” is characterized by a sudden onset of high-grade fever, severe headache, pain behind the eyes, nausea, vomiting, rash and a low total white blood cell count [4]. The hallmark of progression to severe dengue is increased vascular permeability and consequent plasma leakage, leading to circulatory collapse and shock. If shock occurs, it usually takes place after 2 to 6 days of fever [4]. Signs of severe dengue include circulatory compromise or shock, altered mental status, bleeding and unusual manifestations (such as hepatic damage, cardiomyopathy, encephalopathy and encephalitis). Nonetheless, the sensitivity, specificity, and positive and negative predictive value of clinical symptoms and signs in the diagnosis of dengue and severe dengue are far from perfect [4].

Sepsis is a syndrome of systemic manifestations induced by infection with any organism; including bacterial, fungal and viral agents [5–8]. Sepsis can lead to organ dysfunction and mortality. To provide early supportive care and reduce the mortality of sepsis patients, the international Surviving Sepsis Campaign (SSC) in 2004, 2008 and 2012 defined a set of diagnostic criteria for sepsis and severe sepsis, and recommended sepsis bundles for patients with severe sepsis or septic shock [5–7]. For example, crystalloids are recommended as the initial fluid of choice in the resuscitation of severe sepsis and septic shock, based on the absence of any clear benefit following the administration of colloid solutions compared to crystalloid solutions [7].

Sepsis can be caused by dengue virus infection [5–7]; however, many randomized clinical trials of management of adult sepsis patients have not included individuals with dengue. Furthermore, optimal fluid management of dengue patients with sepsis may be different than that of patients with sepsis due to other causes [9, 10]. For example, intravenous colloids are recommended for early resuscitation of patients with dengue shock, particularly if no improvement is observed after rapid volume replacement with 10–20 ml/kg/hour of crystalloid solution [4]. In adult patients who have multiple chronic diseases, dengue infection is associated with high mortality [11, 12]. A recent study reported that 73% of dengue deaths (232/320) in Malaysia in 2013–2014 [13] and 67% of dengue deaths (103/154) in Thailand in 2015 occurred in adult patients (age>15 years) [14]. Therefore, it is essential to gain a better understanding of sepsis in adult patients caused by dengue virus. Here, we report the prevalence, clinical manifestations, diagnosis, management and outcomes of dengue patients who were enrolled into a prospective sepsis study in Thailand (NCT02217592) and were retrospectively diagnosed with dengue.

## Material and methods

### Study design and study site

We conducted a prospective observational (non-interventional) study of community-acquired sepsis and severe sepsis in Sunpasithiprasong Hospital, Ubon Ratchathani province, northeast Thailand. Thailand is an upper-middle income country, spending $264 on health per capita in 2013 [15]. Ubon Ratchathani is the largest province in northeast Thailand with a population of 1.8 million, covers an area of 16,113 km^2^, and is bordered by Cambodia to the south and Laos to the east. Sunpasithiprasong Hospital is a public tertiary-care hospital with 1,200 non-ICU beds and 220 ICU beds, providing care to people living within its catchment area and acting as a referral hospital to smaller district hospitals. Severely ill patients presenting to district hospitals are often referred to Sunpasithiprasong Hospital, which is equipped with microbiology facilities and ICUs. Dengue is generally diagnosed by attending physicians based on clinical manifestations [4] with or without results of diagnostic tests for dengue. Dengue rapid test for the detection of NS1, and IgM and IgG antibodies are usually performed if attending physicians suspect dengue.

### Study participants

We prospectively enrolled adult patients (age≥18 years) who were admitted with a primary diagnosis of suspected or documented infection made by the attending physician, were within 24 hours of hospital admission, and had at least three sepsis diagnostic criteria documented in the medical record. We used 19 variables which were consolidated from the 22 variables proposed as diagnostic criteria for sepsis by SSC 2012 [7], and included ‘low oxygen saturation (SpO_2_<95%)’ (S1 Table). This variable was added because ‘oxygen saturation determined by pulse oximetry’ is recommended by World Health Organization guidelines for limited-resource settings [16]. Altered mental status was defined as a Glasgow Coma Scale (GCS) <15, or <10 if intubated (<10T). We excluded patients who were suspected of having hospital-acquired infections determined by the attending physician, had a hospital stay within 30 days prior to this admission, or were transferred from other hospitals with a total duration of hospitalisation >72 hours.

The study team of trained research nurses sequentially screened all medical patients admitted with a primary diagnosis of infection by conducting ward rounds and reviewing admission logs in the emergency room (ER), medical wards and medical ICUs in the morning and in the afternoon of every working day. Nurses in the ER also notified the study team directly about potentially eligible patients. The presence or absence of diagnostic variables of sepsis used to determine eligibility for the study was determined only from the data documented in the medical charts. If data were not documented in the medical charts, this was noted as ‘not documented (N/D)’. Blood was drawn from all patients at the time of enrolment, and serum and plasma samples were frozen at -80°C.

### Study team point-of-care assessments

At the time of enrolment, every patient was evaluated by the study team at the bedside using four point-of-care assessments: a whole blood lactate RDT (Lactate Pro 2, Arkray Global Business Inc., Australia), a whole blood glucose RDT (ACCU-CHECK Performa, Roche Diagnostic, Germany), pulse oximetry (Nellcor N-65, Covidien plc., Ireland) and GCS. The results were reported to the attending physicians. The study did not involve any clinical interventions; all treatment was provided by the attending physicians and their medical teams. The 28-day vital status was evaluated via telephone contact if subjects were no longer hospitalised and had been discharged alive.

We conducted the study in full compliance with the principles of good clinical practice (GCP), and the ethical principles of the Declaration of Helsinki. The study protocol and related documents were approved by Sunpasithiprasong Hospital Ethics Committee (039/2556), the Ethics Committee of the Faculty of Tropical Medicine, Mahidol University (MUTM2012-024-01), the University of Washington Institutional Review Board (42988) and the Oxford Tropical Research Ethics Committee, University of Oxford (OXTREC172-12). Written informed consent was obtained from all participants prior to the enrolment. The registry number of the main study is NCT02217592. The main prospective sepsis study started in March 2013, and was completed in January 2017.

### PCR assays for dengue viruses

Frozen serum samples collected on enrolment from patients presenting during the most recent rainy season (June to December 2015) were subsequently tested for dengue virus at the Armed Forces Research Institute of Medical Sciences, Bangkok, Thailand. The hemi-nested reverse transcriptase polymerase chain reaction (RT-PCR) was conducted as described previously and identified serotypes DENV-1 to -4 [17]. We reported the outcomes of adult dengue patients prior to the completion of the entire sepsis study because we believe that our study would have clinical implications for the management of patients presenting with sepsis in tropical countries where dengue is endemic.

### Statistical analysis

We compared clinical manifestations and management between adult dengue patients who survived and those who died. Dengue was diagnosed by PCR assays as described above. The Fisher’s exact test and Mann-Whitney test were used to compare categorical and continuous variables between groups, respectively. Interquartile ranges (IQRs) are presented in terms of 25^th^ and 75^th^ percentiles. We evaluated the association between factors and 28-day mortality using univariable and multivariable Firth logistic regression models [18]. Firth logistic regression model was used because a few potentially important variables had rare variants. The final multivariable logistic regression models were developed using purposeful selection as described by Hosmer and Lemeshow [19]. In short, all variables significant in the univariable analysis at p value <0.20 were evaluated. Non-significant variables were deleted one at a time, and also re-tested by adding back to the initial multivariable model if they were previously deleted. Due to the limited sample sizes, only three variables were kept in the final multivariable model. As we observed that dengue was not diagnosed in all dengue patients who died, we hypothesized that some clinical presentations might decrease suspicion for dengue. We also evaluate whether those dengue patients would be classified as sepsis based on the most up-to-date diagnostic criteria for sepsis, Sepsis-3 [8]. Therefore, we additionally evaluated clinical presentations and Sequential Organ Failure Assessment (SOFA) scores among dengue patients who were diagnosed and misdiagnosed by attending physicians [20]. A score of zero was assigned to missing SOFA scores for individual organ systems. We defined severe sepsis as SOFA score ≥2 in the setting of sepsis. All analyses were performed using STATA version 14.0 (StataCorp, College Station, TX, USA). The final database and the data dictionary are publicly available online (https://figshare.com/s/a31da6384a6a50c99464).

### Role of the funding source

All study procedures, data collection, data analyses, data interpretation, and writing of the report were performed solely by the participating authors without the sponsors’ involvement. Full access to data was granted to the corresponding author. All authors participated in the study design or analysis, and approved the submission of the manuscript.

## Results

### Baseline patient characteristics

From 1^st^ June to 31^st^ December 2015, 874 adult patients with community-acquired sepsis were enrolled. Stored serum was available and tested using a dengue PCR assay on 866 patients (99%). Dengue PCR assays were positive in 126 patients (15%), of which 2 (2%), 12 (10%), 24 (19%) and 88 (70%) were positive for DENV-1, DENV-2, DENV-3 and DENV-4, respectively.

Table 1 and S2 Table show the characteristics of the 126 adult patients with sepsis and dengue infection included in the analysis. 59 (47%) were male and the median age was 27 years (IQR 21–39 years, range 18–92 years). Underlying diseases were observed in 18 patients (14%); diabetes was most frequent (8 patients, 6%). Most patients had diagnostic criteria of ‘fever or hypothermia’, ‘heart rate >90/min’, ‘tachypnoea’ and ‘leukocytosis, leukopenia or immature forms >10%’. A total of 70 patients (56%) had platelet counts <100,000/μL.

**Table 1:** Baseline characteristics of 126 adult patients with sepsis and dengue infection.

Characteristics	Survived (n = 121)	Died (n = 5)	P values
**Gender, male**	55 (45%)	4 (80%)	0.19
**Median age (interquartile range, range), years**	26 (21–35, 18–92)	64 (63–74, 51,75)	<0.001
**Prior or pre-existing conditions**	14 (12%)	4 (80%)	0.001
Diabetes	5 (4%)	3 (60%)	0.002
Hypertension	4 (3%)	2 (40%)	0.02
Chronic kidney disease	4 (3%)	2 (40%)	0.02
Others ^a^	6 (5%)	0 (0%)	>0.99
**Presence of diagnostic criteria for sepsis** ^b^			
Fever (>38.3°C) or hypothermia (<36°C)	109 (90%)	5 (100%)	>0.99
Heart rate >90/min	110 (91%)	5 (100%)	>0.99
Tachypnoea (Respiratory rate >20/min)	88 (73%)	5 (100%)	0.33
Altered mental status (Glasgow Coma Scale <15 or <10T)	5 (4%)	5 (100%)	<0.001
Significant edema or positive fluid balance ^c^	2/66 (3%)	0/3 (0%)	>0.99
Hyperglycaemia in the absence of diabetes	28 (23%)	2 (40%)	0.59
Leukocytosis, leukopenia or immature forms >10%	81 (67%)	5 (100%)	0.18
Arterial hypotension	32 (26%)	4 (80%)	0.02
Low oxygen saturation (SpO_2_ <95%) or required mechanical ventilation ^c^	2/118 (2%)	3 (60%)	<0.001
Creatinine increase > 0.5 mg/dL ^c^	2/33 (6%)	2/4 (50%)	0.05
Coagulation abnormalities (INR >1.5 or aPTT >60s) ^c^	15/26 (58%)	3/5 (60%)	>0.99
Thrombocytopenia (platelet count <100,000/μL)	68 (56%)	2 (40%)	0.67
Hyperbilirubinaemia (plasma total bilirubin >4mg/dL) ^c^	3/104 (3%)	0/3 (0%)	>0.99
Hyperlactataemia (>1mmol/L)	88 (73%)	4 (80%)	>0.99
Decreased capillary refill or mottling ^c^	1/11 (9%)	1/1 (100%)	0.17

^a^ Thalassemia (n = 2), Pregnancy (n = 1), HIV infection (n = 1) and chronic obstructive pulmonary disease (n = 1).

^b^ Adapted from Dellinger et al, Surviving Sepsis Campaign: International Guideline for Management of Severe Sepsis and Septic Shock: 2012. Diagnostic criteria were based on data documented in medical charts prior to enrolment, laboratory tests performed on admission by attending physicians and point-of-care assessments performed by study team immediately following enrolment.

^c^ Denominators varied owing to missing data

### Outcomes

Of 126 patients adult patients with sepsis and dengue infection included in the analysis, 5 (4%) died within 28 days of admission. All five patients had a history of fever prior to admission. Three (60%) died within the first 3 days of admission, and the other two died on 6 and 16 days of admission. The first fatal case (64 year-old male with underlying diabetes and hypertension) presented with profound shock. He received fluid resuscitation and adrenergic agents. On day 2, he developed focal convulsions and upper gastrointestinal bleeding. He was treated for septic shock and disseminated intravascular coagulopathy. He had sudden cardiac arrest and died on day 3. The second fatal case (63 year-old female) presented with profound shock. She was treated for septic shock. Her blood pressure recovered after fluid resuscitation and adrenergic agents. On day 2, she had hypotension again (blood pressure 83/46 mmHg), developed sudden cardiac arrest and died. The third fatal case (74 year-old male) presented with alteration of conscious. His Glasgow Coma Score was 12 on enrolment and continuously declined. His cerebrospinal fluid showed the profile of viral meningitis. He had multiple peaks of fever and had no arterial hypotension over the admission period. He died on day 6. The fourth fatal case (51 year-old male) presented with profound shock and was treated for septic shock. He received fluid resuscitation and adrenergic agents. On day 2, he had hypotension again (blood pressure 83/47 mmHg), developed sudden cardiac arrest and died. The fifth fatal case (75 year-old male) had non-productive cough for 3 weeks before he developed high grade of fever for 7 days. On the admission day, he developed severe dyspnea and presented to the hospital with profound shock and pneumonia. He was intubated and required mechanical ventilation since admission. He also had sputum culture positive for *Burkholderia pseudomallei* and was treated for melioidosis with septic shock. He developed bilateral pneumothorax on day 8 and died on day 16. For those who survived, the median duration of hospital stay was 4 days (interquartile range 3–5 days, range 1–57 days). The patient who stayed in the hospital for 57 days had respiratory failure requiring mechanical ventilation. He later developed hospital-acquired pneumonia. He required a tracheostomy and long duration of intravenous antibiotics prior to hospital discharge.

### Hemodynamic and volume management

We recorded components of sepsis management during the first three days of admission and found that bolus fluid and adrenergic agent administration were recorded in 24 (19%) and 14 (11%) patients, respectively (Table 2). All four patients who had hypotension on admission primarily received crystalloids and eventually received colloid (n = 3), packed red blood cells (n = 1) or fresh frozen plasma (n = 2) (no more than 500 ml/patient) as a part of intravenous fluid resuscitation during the first 24 hours (the total volume of fluid received ranged from 4 to 6 litres/patient). None of the four patients received active haematocrit monitoring.

**Table 2:** Management and diagnoses of 126 adult patients with sepsis and dengue infection.

Variables	Survived (n = 121)	Died (n = 5)	P values
**Management within 3 days of admission** ^a^			
Bolus intravenous fluid administration	20 (17%)	4 (80%)	0.005
Bolus intravenous fluid amount (ml); median (IQR; number of patients with data available)	1,000 (500–2,000; n = 19)	2,050 (1,050–2,600; n = 4)	0.16
Adrenergic agent administration	10 (8%)	4 (80%)	<0.001
**Primary diagnosis made by attending physicians**			
Dengue infection	84 (69%)	0 (0%)	0.003
Acute febrile illness or systemic infection	35 (39%)	1 (20%)	>0.99
Septic Shock	7 (6%)	3 (60%)	0.003
Sepsis	2 (2%)	1 (20%)	0.12
Pneumonia	6 (5%)	2 (40%)	0.03
Others ^b^	7 (6%)	1 (20%)	0.28
**Final diagnosis made by attending physicians** ^c^			
Dengue infection	96 (79%)	0 (0%)	0.001
Septic Shock	8 (7%)	4 (80%)	<0.001
Sepsis	5 (4%)	0 (0%)	>0.99
Pneumonia	3 (2%)	3 (60%)	0.001

^a^ Based on data documented in medical charts prior to enrolment, laboratory tests performed on admission by attending physicians, point-of-care assessments and daily assessments for 3 consecutive days performed by study team.

^b^ Included acute gastroenteritis, bronchitis, cellulitis, infected continuous ambulatory peritoneal dialysis, malaria, melioidosis and urinary tract infection for those who survived (n = 7), and acute meningitis for the one who died (n = 1).

^c^ Final diagnosis was based on a record of International Classification of Disease (ICD) 10th made by attending physicians. Dengue infection included ICD10 code of A90 and A91. Septic shock included ICD10 of R57.2, R65.1, and R65.21. Sepsis included ICD10 code of A41, A41.9 and R65. Pneumonia included ICD10 code of J18.

### Clinician diagnosis of dengue

Overall, we found that attending physicians suspected dengue infection on admission in 84 patients (67%), and recorded dengue infection as the final diagnosis in 96 patients (76%). The other common final diagnoses by clinicians were septic shock (12 patients; 10%), pneumonia (6 patients; 5%) and sepsis (5 patients; 4%) (Table 2). All three patients who had profound shock on admission and died within the first three days of admission were diagnosed as septic shock. All three of them provided a history of fever for two days prior to hospital admission but none had fever on admission (body temperature ranged from 37.0 to 37.7°C), and their pulse pressures were not lower than 20 mmHg (S2 Table). We also observed that complete blood counts on admission of those who died were not the classic abnormalities seen in dengue patients (S2 Table).

### Factors associated with clinician diagnosis of dengue

Of 126 dengue patients with sepsis, 89 (71%) patients had a SOFA score ≥2 on admission. We found that SOFA score on enrolment was not associated with primary diagnosis of dengue made by attending physicians (S3 Table). However, those who had clinical manifestations consistent with classic dengue findings of three to five days of fever, leukopenia, high percentage of lymphocytes, high haematocrit, and thrombocytopenia were significantly more likely to have a primary diagnosis of dengue made by attending physicians (S3 Table). In addition, creatinine concentrations were significantly lower in patients diagnosed with dengue.

### Factors associated with death

In univariable analysis, older age and having pre-existing conditions were associated with mortality (Table 1). Signs of severe dengue including altered mental status, arterial hypotension and low oxygen saturation or requirement for mechanical ventilation were strongly associated with mortality (Table 1). Misdiagnosis of dengue infection as the etiology of sepsis was also significantly associated with mortality outcome. None of the five patients who died were diagnosed with dengue infection by attending physicians (Table 2).

In multivariable analysis, there was a trend showing that older age (age ≥60years), low oxygen saturation or requirement for mechanical ventilation, and misdiagnosis of dengue infection were associated with mortality (Table 3).

**Table 3:** Factors associated with mortality.

Factors	Crude odds ratio (95% confidence interval)	P values	Adjusted odds ratios (95% confidence interval)	P values
Older age (age ≥60years)	31.9 (4.5–224.6)	0.001	7.9 (0.7–89.7)	0.09
Low oxygen saturation (SpO_2_ <95%) or requirement for mechanical ventilation ^a^	65.2 (8.4–506.9)	<0.001	29.0 (2.3–371.1)	0.01
Misdiagnosis of dengue infection ^b^	41.6 (2.2–777.8)	0.01	17.3 (0.6–531.8)	0.10

N/A = Not applicable.

^a^ From the pre-transfer period until enrolment

^b^ Final diagnosis did not include dengue infection (ICD10 code of A90 or A91).

## Discussion

Our findings suggest that a sizeable proportion of adult patients with sepsis admitted to a Thai referral hospital have dengue virus infection. In addition, while clinicians frequently diagnosed dengue in surviving patients presenting with sepsis, this infection was not considered in severely ill patients who subsequently died. Overall, clinicians were also less likely to diagnose dengue infection when duration of symptoms was shorter, and white blood cell counts, platelet counts and creatinine levels were higher. These data raise concerns about the burden, management and prevention of dengue. Recent spatial modelling work estimates there to be 390 million dengue infections per year worldwide, of which 96 million are clinically apparent [3]. The Global Burden of Disease Study reports that about 9,110 deaths were attributable to dengue in 2013 [21]. The model estimated that 46% of deaths attributable to dengue occurred in adults (age ≥15 years) [22]. Overall, this could be an underestimate due to under-recognition and under-reporting of dengue, and the limited availability of national statistics in developing tropical countries [22]. Our study suggests that septic patients with dengue in a tropical developing country can be misdiagnosed and that national statistics for dengue deaths in Thailand, particularly in adult patients [14], could be substantially under-reported.

According to the most up-to-date diagnostic criteria for sepsis, Sepsis-3 [8], patients with infectious disease-related clinical presentations and Sequential Organ Failure Assessment (SOFA) score of 2 points or more should be diagnosed as sepsis. This definition replaces the previous term “severe sepsis” and is generated in order to provide the early recognition and accurate burden quantification of sepsis worldwide [8]. Importantly, these revised sepsis definitions were not developed using cohorts of patients with dengue infection. Severe dengue patients may have thrombocytopenia, shock requiring adrenergic agents, or alteration of conscious leading to a total SOFA score of ≥2. Regardless of infectious etiology, these findings are likely to suggest significant organ insufficiency; therefore, dengue patients who fulfil the sepsis definition should also be simultaneously diagnosed as sepsis and noted to attending physicians for prompt management [8].

All sepsis patients should be evaluated in order to identify the causative organism [7]. The SSC bundles recommend that sepsis patients should receive blood cultures prior to administration of antibiotics within 3 hours [7]. Our study suggests that a considerable proportion of sepsis in tropical countries may be caused by dengue, particularly in adult patients. The dengue virus is not detected by blood culture. Furthermore, the clinical presentation of dengue is non-specific, and misdiagnosis of dengue is common [14]. Although the presence of leucopenia and thrombocytopenia should increase clinical suspicion for dengue, many severe dengue patients do not present with those clinical manifestations [4, 14]. Paediatric patients with severe dengue without leukocytopenia or thrombocytopenia have been described [23–25]. Our study shows that this may also occur in adult dengue patients. The WHO guideline also advises against relying on the presence of bleeding or a specific platelet cutoff to diagnose dengue and severe dengue [4]. All fatal cases in our study presented with alteration of consciousness (Table 1), which could be considered as lethargy within warning signs suggested by the WHO guideline (which include abdominal pain or tenderness, persistent vomiting, clinical fluid accumulation, mucosal bleed, lethargy, restlessness and liver enlargement) [4]. Mental status is also included in the qSOFA score, which is newly recommended in the Sepsis-3 definitions as simple clinical criteria to screen patients for sepsis [8]. Therefore, in order to rapidly detect dengue in patients presenting with sepsis, we propose that rapid diagnostic tests for dengue should be considered in the bundle of sepsis management in tropical countries, if resources are available.

Optimal management of sepsis caused by dengue viruses and bacteria may not necessarily be similar, underscoring the importance of early diagnosis of the etiology of sepsis. Colloids are not recommended during the initial resuscitation of septic shock, as there is no evidence to suggest that they are better than crystalloids [7]. However, based on limited evidence of randomized controlled trials in paediatric dengue patients, colloids are recommended for early resuscitation of patients with dengue shock [4]. It is common for paediatric patients with dengue shock to receive up to 20 mL/kg of colloid during fluid resuscitation [26]. It is possible that colloids might be useful for initial fluid resuscitation of adult dengue patients with hypotension, and further randomized controlled trials for fluid management and adrenergic agent administration in adult dengue patients are needed [27, 28]. In addition, active haematocrit monitoring should be performed to assist in titrating the fluid and blood product administration among sepsis patients caused by dengue virus. SSC recommends red blood cell transfusion when the hemoglobin concentration decreases to <7.0 g/dL to target a hemoglobin concentration of 7.0 to 9.0 g/dL in adults with sepsis [7]. However, for dengue patients, red blood cell transfusion should be considered when rapid volume replacement is provided, haematocrit falls, and bleeding is suspected [4]. Although currently there is no evidence supporting other specific treatments for severe dengue, this may change in the future [28, 29].

There is growing evidence of increasing dengue incidence in older age groups, and the reason for this change is still largely unknown [30, 31]. Our study findings also support previous reports that severe dengue in adult patients with chronic diseases are associated with mortality [11, 12]. Dengue with central nervous system involvement, including aseptic meningitis and encephalitis, is not uncommon and is associated with mortality [32, 33]. Bacterial co-infections in dengue patients are uncommon but important, as they are also associated with mortality [34, 35].

Vector control and early diagnosis of dengue prior to the development of dengue shock syndrome should be considered as one of the main methods to reduce mortality caused by dengue in adult population. Four patients who died of dengue in our study had profound shock on admission, and aggressive fluid resuscitation after the onset of profound shock might not be able to reverse the established organ failure. As shock usually occurs between day 3 to 7 of fever, close follow-up of all suspected and confirmed dengue patients could facilitate prompt management even in adult patients [4]. Vector control is also important and is closely linked to diagnosis [36]. Implementation of vector control measures can prevent additional dengue cases, and consequently reduce mortality caused by dengue in the population [36].

This study has some limitations. First, the high prevalence of dengue in adult sepsis patients in this study (15%) could be associated with the high incidence rate of dengue in Thailand in 2015 [14]. Second, the study might in fact underestimate the proportion of dengue because some dengue patients might have false negative dengue PCR assays. Third, the study did not compare management and outcome of patients with sepsis due to dengue and that of patients with sepsis due to other causes. Fourth, the sample size of dengue patients in the analysis was small and some variables had rare variants; therefore, the association between risk factors and mortality should be interpreted with caution. Fifth, the true SOFA score could be higher if blood gases and other blood tests were measured in all patients; nonetheless, our results represent the real situation in an upper middle-income country in Southeast Asia. Sixth, the prospective study was designed based on SSC 2012 rather than Sepsis-3 criteria. Further studies of application and utility of Sepsis-3 criteria in resource-limited settings are still required.

## Conclusions

We show that dengue is a frequent etiology of sepsis in adults in northeast Thailand and that the diagnosis of dengue may not be made in patients with severe illness who die of shock. Current sepsis management guidelines are generally not derived from studies of septic patients with dengue infection and optimal management of dengue may differ from that of other causes of sepsis. We propose that our findings provide a starting point for a wider discussion about the impact of dengue in adults precenting with sepsis and inform the goal of improving diagnosis, treatment and prevention of dengue in the adult population.

## Supporting information

S1 Table Diagnostic criteria for sepsis documented in the medical charts at the time of enrolment. (DOCX)

S2 Table Baseline characteristics of 126 adult patients with sepsis and dengue infection by outcome. (DOCX)

S3 Table Baseline characteristics of 126 adult patients with sepsis and dengue infection by primary diagnosis. (DOCX)
