# Supplementary material for: Management and outcomes of severe dengue patients presenting with sepsis in a tropical country
Source: PLoS One. 2017 Apr 24;12(4):e0176233. doi: 10.1371/journal.pone.0176233 (PMC5402971; doi:10.1371/journal.pone.0176233)
Supplement: S1 Table — (DOCX) [file pone.0176233.s001.docx]

**S1 Table. Diagnostic criteria for sepsis documented in the medical charts at the time of enrollment**

| **Infection, documented or suspected, and some of the following:** |
| --- |
| **General variables** |
| Fever or hypothermia (body temperature >38.3^o^C or <36^o^C) **^a^** |
| Heart rate >90/min^-1^ |
| Tachypnoea (respiratory rate >20/min^-1^) |
| Altered mental status (Glasgow Coma Scale <15 or <10T) ^b^ |
| Significant oedema or positive fluid balance (20 mL/kg over 24 hr) |
| Hyperglycaemia (plasma glucose >140 mg/dL) in the absence of diabetes |
| **Inflammatory variables** |
| Leukocytosis (WBC count >12,000 u/L), Leukopenia (WBC count <4,000 u/L), or immature forms >10% **^c^** |
| Plasma C-reactive protein more than two SD above the normal value |
| Plasma procalcitonin more than two SD above the normal value |
| Haemodynamic variables |
| Arterial hypotension (SBP <90 mmHg, MAP <70 mmHg or an SBP decrease >40 mmHg) |
| **Organ dysfunction variables** |
| Low oxygen saturation determined by pulse oximetry (SpO_2_ <95%) ^d^ |
| Arterial hypoxaemia (PaO_2_/FiO_2_ <300) |
| Acute oliguria (urine output <0.5 mL/kg/hr for at least 2 hrs) ^e^ |
| Creatinine increase >0.5 mg/dL |
| Coagulation abnormalities (INR >1.5 or aPTT >60s) |
| Ileus (absent bowel sounds) |
| Thrombocytopenia (platelet count <100,000 u/L) |
| Hyperbilirubinaemia (plasma total bilirubin >4 mg/dL) |
| **Tissue perfusion variables** |
| Hyperlactataemia (>1 mmol/L) |
| Decreased capillary refill or mottling |

Adapted from Dellinger et al, Surviving Sepsis Campaign: International Guideline for Management of Severe Sepsis and Septic Shock: 2012

**^a^** Variables fever and hypothermia were consolidated into a single variable.

**^b^** Glasgow Coma Scale <15 or <10T was defined for the altered mental status variable.

**^c^** Variables Leukocytosis, Leukopenia and immature forms >10% were consolidated into a single variable.

**^d^** Variable Low oxygen saturation determined by pulse oximetry (SpO_2_ <95%) was added.

**^e^** There is a condition of ‘despite adequate fluid resuscitation’ for this criterion in the Surviving Sepsis Campaign 2012 diagnostic criteria for severe sepsis.
