# Supplementary material for: Management and outcomes of severe dengue patients presenting with sepsis in a tropical country
Source: PLoS One. 2017 Apr 24;12(4):e0176233. doi: 10.1371/journal.pone.0176233 (PMC5402971; doi:10.1371/journal.pone.0176233)
Supplement: S2 Table — (DOCX) [file pone.0176233.s002.docx]

**S2 Table. Baseline characteristics of 126 adult patients with sepsis and dengue infection by outcome**

| **Characteristics ^a^** | **Survived**  **(n=121)** | **Died**  **(n=5)** | **P values ^b^** |
| --- | --- | --- | --- |
| Duration of symptoms (days) | 4 (3-5) | 6 (2-30) | 0.25 |
| **Vital signs** |  |  |  |
| Body temperature (°C) | 38 (37.3-38.7) | 37.2 (37.1-37.5) | 0.08 |
| Heart rate (bpm) | 93 (82-101) | 119 (117-122) | 0.04 |
| Systolic blood pressure (mmHg) | 116 (104-125) | 92 (80-102) | 0.07 |
| Diastolic blood pressure (mmHg) | 68 (61-73) | 51 (48-56) | 0.03 |
| Pulse pressure | 46 (37-55) | 44 (36-46) | 0.58 |
| **Complete blood count** |  |  |  |
| White blood cell (/μL) | 3620 (2630-5690) | 16610 (14450-20760) | 0.05 |
| Percentage of neutrophils (%) | 60 (44-74) | 92 (75-93) | 0.009 |
| Percentage of lymphocytes (%) | 32 (17-41) | 6 (4-16) | 0.006 |
| Hematocrit (%) | 39.8 (35.3-43.0) | 29.2 (23.3-33.7) | 0.002 |
| Platelet count (/μL) | 74,000  (26,000-170,000) | 182,000  (86,000-183,000) | 0.15 |
| **Dengue virus** |  |  |  |
| Type I | 1 (1%) | 1 (20%) | 0.10 |
| Type II | 12 (10%) | - |  |
| Type III | 23 (19%) | 1 (20%) |  |
| Type IV | 85 (70%) | 3 (60%) |  |

**^a^** Based on data documented in medical charts on admission. Data are presented as median and interquartile range.

**^b^** Based on univariable analyses using Fisher exact test and Mann-Whitney test.
