# Supplementary material for: Management and outcomes of severe dengue patients presenting with sepsis in a tropical country
Source: PLoS One. 2017 Apr 24;12(4):e0176233. doi: 10.1371/journal.pone.0176233 (PMC5402971; doi:10.1371/journal.pone.0176233)
Supplement: S3 Table — (DOCX) [file pone.0176233.s003.docx]

**S3 Table. Baseline characteristics of 126 adult patients with sepsis and dengue infection by primary diagnosis**

| **Characteristics ^a^** | **Patients with primary diagnosis of dengue infection (n=84)** | **Patients with primary diagnosis of other diseases (n=42)** | **P values ^b^** |
| --- | --- | --- | --- |
| Duration of symptoms (days) | 4 (3-5) | 2 (2-4) | <0.001 |
| **Vital signs** |  |  |  |
| Body temperature (°C) | 38.0 (37.5-38.7) | 37.9 (37.0-38.6) | 0.29 |
| Heart rate (bpm) | 90 (80-100) | 99 (87-112) | 0.04 |
| Systolic blood pressure (mmHg) | 116 (105-124) | 111 (97-128) | 0.19 |
| Diastolic blood pressure (mmHg) | 70 (61-76) | 65 (58-70) | 0.06 |
| Pulse pressure | 47 (38-55) | 45 (37-53) | 0.68 |
| **Complete blood count** |  |  |  |
| White blood cell (/μL) | 3,140 (2,495-4,225) | 7,115 (4,790-15,250) | <0.001 |
| Percentage of neutrophils (%) | 54% (41-64%) | 79% (70-85%) | <0.001 |
| Percentage of lymphocytes (%) | 35% (27-45%) | 14 (9-20%) | <0.001 |
| Hematocrit (%) | 41.6 (36.8-44.0) | 34.7 (29.1-39.3) | <0.001 |
| Platelet count (/μL) | 58,500  (21,500-100,000) | 179,500  (130,000-219,000) | <0.001 |
| **Blood chemistry** |  |  |  |
| Bilirubin (mg/dL; number of patients with data available) | 0.5 (0.3-0.8; n=72) | 0.6 (0.4-0.9; n=34) | 0.08 |
| Creatinine (mg/dL; number of patients with data available) | 0.8 (0.6-1.0; n=80) | 0.9 (0.7-2.0; n=42) | 0.001 |
| Glasgow Coma Scale score | 15 (15-15) | 15 (15-15) | 0.02 |
| Total SOFA score **^c^** | 3 (2-4) | 3 (1-4) | 0.97 |

**^a^** Based on data documented in medical charts on admission. Data are presented as median and interquartile range.

**^b^** Based on univariable analyses using Fisher exact test and Mann-Whitney test.

**^c^** Only two patients had blood gas performed on admission.
